# No Longer Cutaneous T-cell Lymphoma Dermal Nodule

**DOI:** 10.7759/cureus.50796

**Published:** 2023-12-19

**Authors:** Miranda L Yousif, Xiangfeng Zhao, Debora Andrews

**Affiliations:** 1 Medicine, University of Arizona College of Medicine - Phoenix, Phoenix, USA; 2 Pathology, Carl T. Hayden Veterans' Administration Medical Center, Phoenix, USA; 3 Dermatology, Carl T. Hayden Veterans' Administration Medical Center, Phoenix, USA

**Keywords:** b lymphocytes, t lymphocytes, cutaneous t cell lymphoma, s100, primary cutaneous cd4+ small/medium lymphoproliferative disorder

## Abstract

Primary cutaneous CD4+ small and medium pleomorphic T-cell lymphoproliferative disorder (PCSM-LPD) is a rare and typically asymptomatic proliferation of CD3+/CD4+ small and medium pleomorphic T-cells. In this case report, we share the details of a 41-year-old male presenting with a two-centimeter soft, mobile forehead nodule that was determined by clinical symptoms, histology, and immunostaining to be PCSM-LPD. We would like to emphasize the clinical resolution that was seen with minimal treatment.

## Introduction

Primary cutaneous CD4+ small and medium pleomorphic T-cell lymphoproliferative disorder (PCSM-LPD) is defined as a rare, benign, usually asymptomatic proliferation of predominantly CD3+/CD4+ small and medium pleomorphic T-cells. In 2016, the World Health Organization changed the classification of this diagnosis from lymphoma to "lymphoproliferative disorder" due to its benign, localized nature and typically excellent prognosis [1].

In this case report, we describe a 41-year-old male patient with no significant past medical history who presented to dermatology with a three-month history of an enlarging two-centimeter soft, mobile nodule on his right forehead. Past skin history was notable only for a right painful wrist furuncle complicated by folliculitis that was drained over 15 years prior. The patient's history suggested no insect bites, immunological diseases, or any drug-inducing factors that may have led to a pseudo-T-cell lymphoma phenomenon [2]. He also denied any systemic or constitutional symptoms, pruritus, or discharge originating from the nodule. The absence of these significant risk factors helped us to narrow down our differential diagnosis, as well [2].

## Case presentation

Upon presentation, it was determined that further testing was needed to identify the pathology of the nodule, in which a punch biopsy and shave biopsy were decided to be the most appropriate initial diagnostic measures. A four-millimeter punch biopsy was obtained from the patient's two-centimeter nodule, located in the right, lateral, high forehead, and a nine-millimeter shave biopsy was obtained from a fleshy skin-colored papule located on his right outer buttock. Results of the shave biopsy ultimately revealed a nevus lipomatosus superficialis, but his punch biopsy suggests a textbook-like diagnosis of PCSM-LPD. 

**Figure 1:**
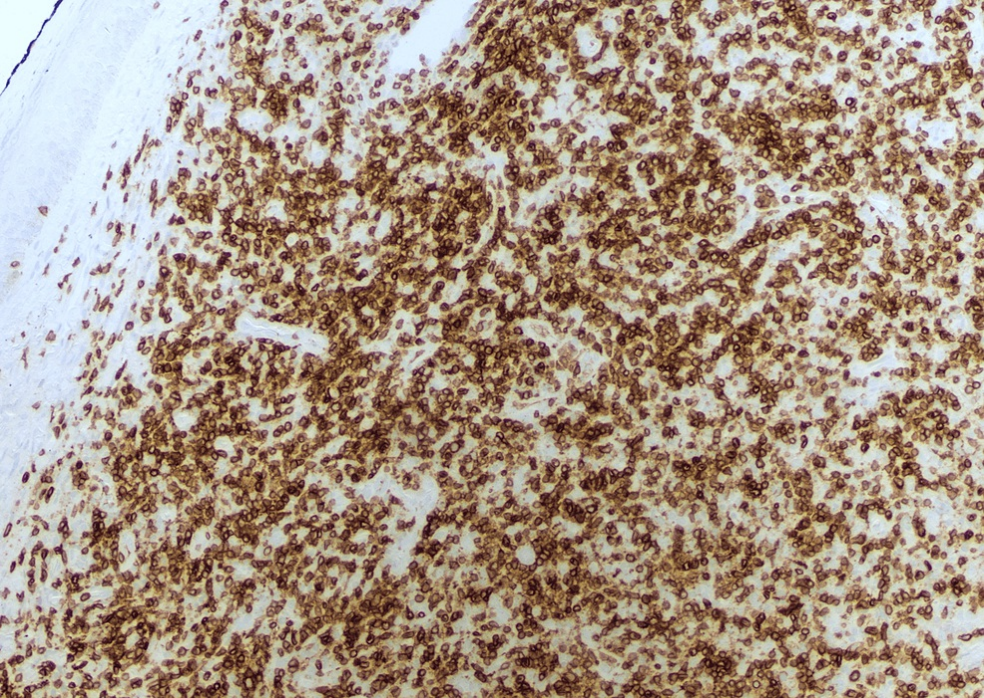
Primary cutaneous CD4+ T-cell lymphoproliferative disorder (immunohistochemistry, magnification 200x) The lymphoma cells are positive for CD3.

**Figure 2:**
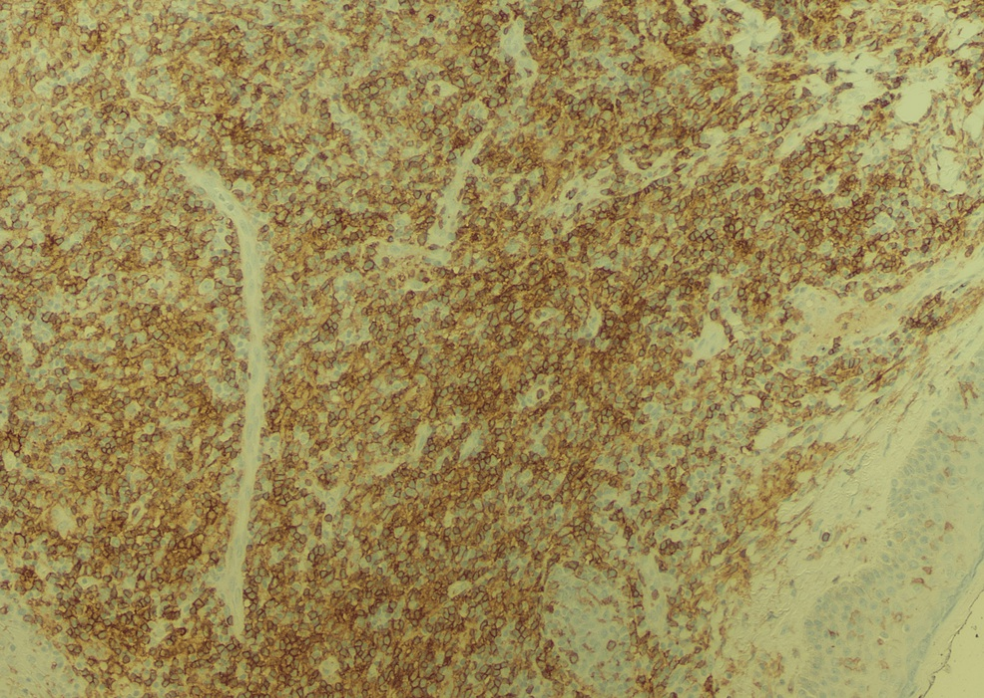
Primary cutaneous CD4+ T-cell lymphoproliferative disorder (immunohistochemistry, magnification 200x) The lymphoma cells are positive for CD4.

Hematoxylin and eosin (H&E) staining of the punch biopsy revealed a subcutaneous dense lymphoid infiltrate of small lymphoid cells with a scant amount of clear cytoplasm and no large atypical cells. The infiltrate was bottom-heavy, extending deep into the subcutaneous fibroadipose tissue with very few mitotic figures seen. A paraffin immunoperoxidase stain revealed a predominance of small T-cells expressing CD2/3/4/5/43+ (see Figures 1-2). 

**Figure 3:**
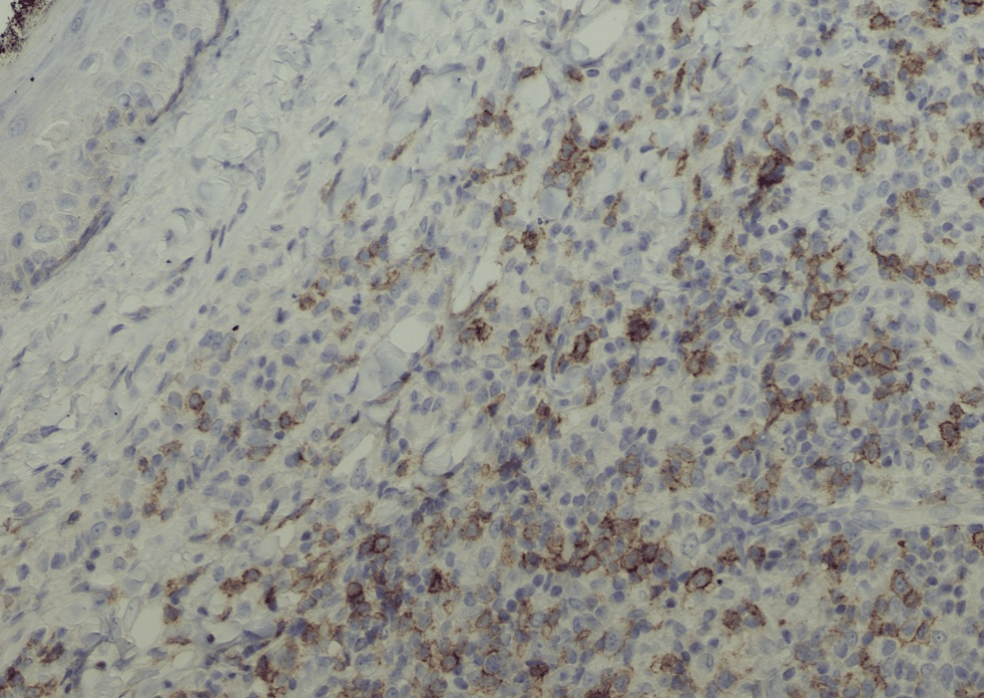
Primary cutaneous CD4+ T-cell lymphoproliferative disorder (immunohistochemistry, magnification 200x) The lymphoma cells are positive for PD1.

**Figure 4:**
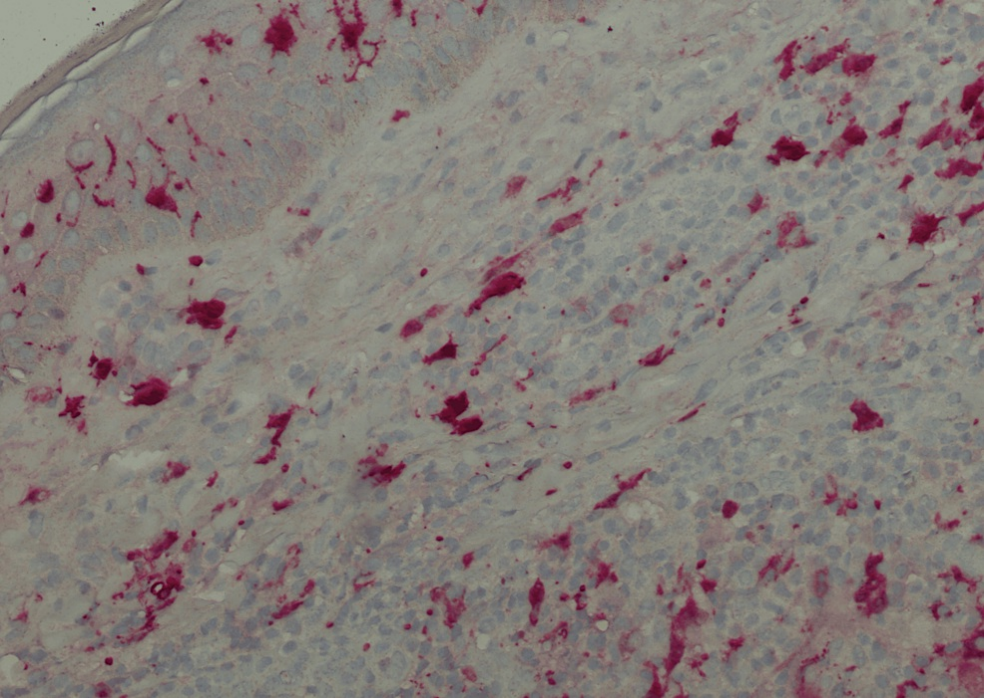
Primary cutaneous CD4+ T-cell lymphoproliferative disorder (immunohistochemistry, magnification 200x) The lymphoma cells are positive for S100.

Staining also revealed PD1+ atypical lymphocytes and S100+ scattered background Langerhans cells (see Figures 3-4). The T-cell population was intermixed with small CD20+ B-cells and CD138+ plasma cells without kappa/lambda restriction, and no epidermotropism was seen.

Molecular studies revealed equivocal T-cell receptor gene rearrangement, indicating a monoclonal T-cell population but no monoclonal immunoglobulin gene rearrangement, further corroborating the monoclonal nature of the T-cell population seen in earlier reviews [3]. Although, we do recognize that these results may be obscured by the polyclonal background. 

From the analysis of the H&E and paraffin immunoperoxidase stains and molecular studies, we diagnosed primary cutaneous CD4+ small/medium T-cell lymphoproliferative disorder, a characteristically rare disorder that is becoming increasingly recognized in the literature. The constellation of findings from staining, molecular studies, and clinical presentation were the most important factors that allowed us to confidently diagnose this condition. 

**Figure 5:**
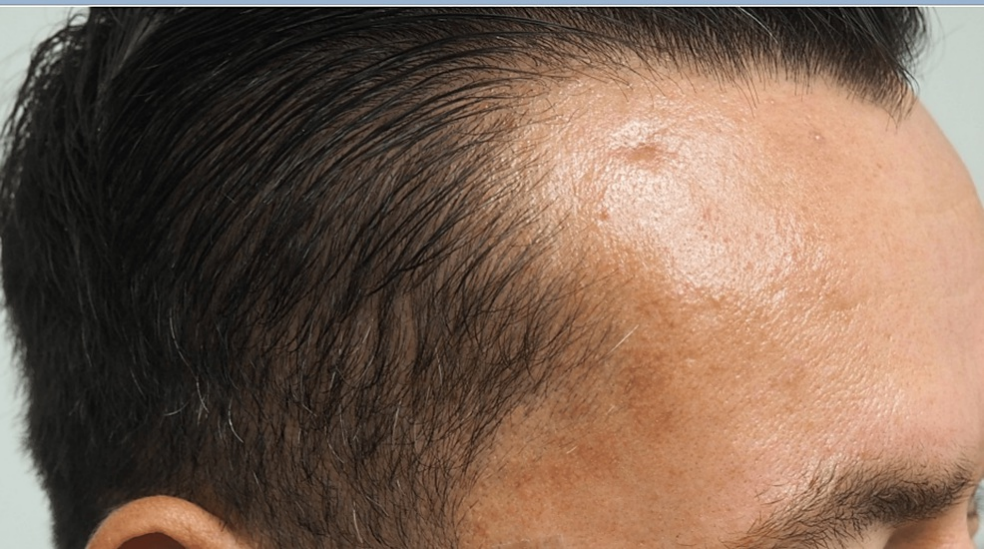
Lateral view photograph of the lesion with clinical resolution following biopsy

**Figure 6:**
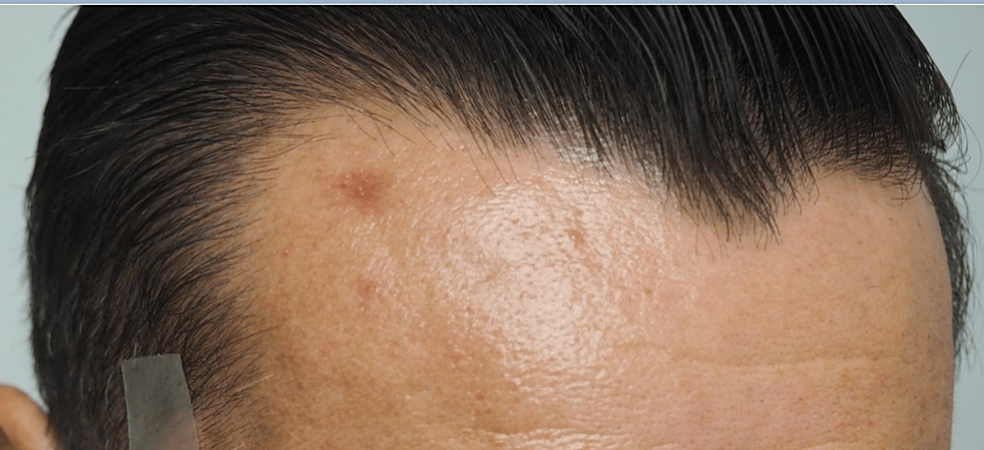
Frontal view photograph of the lesion with clinical resolution following biopsy

For this patient, we determined a punch biopsy would be the most effective method to not only biopsy the lesion but also attempt to remove the entire pathology completely. After punch biopsy, the treated nodule resulted in clinical resolution, as evidenced by the complete flattening of the nodule and surrounding area (see Figures 5-6). 

## Discussion

PCSM-LPD classically presents as a solitary skin plaque or nodule on the face, trunk, or upper extremity, usually present for one to two months prior to presentation [4]. The course of this disease also has shown significant variation, with some cases growing to average lesion sizes within weeks, while others can exhibit slower growth over the years and either persist or spontaneously resolve [5]. Patients typically lack any constitutional or systemic symptoms. The proliferation of small and medium-sized CD3+/CD4+ T-cells is often intermixed with small, reactive cytotoxic CD8+ T-cells, B-cells, plasma cells, and histocytes or multinucleated giant cells all present within the dermis, and the cells typically invade into the subcutis [1].

PCSM-LPD is generally reported to represent only from two to three percent of all primary cutaneous lymphomas [1]. However, in reality, the prevalence may be higher than reported due to the potential misclassification of a characteristically rare condition, suggesting the disease may be underdiagnosed and underreported. Data from some studies reveal a relatively common prevalence of up to 12.5% of all cutaneous T-cell lymphomas seen in some cutaneous lymphoma clinics, second in frequency only to mycosis fungoides [3]. Lesions on the head and neck are typically the most common affected area, with lesions on the trunk being the second most common area [6]. Studies have shown the median age at diagnosis to be between 55-65 years old, but it can present at any age with five-year survival rates of 90%-100% and very rare instances of recurrence, a very favorable prognosis [4,7].

The differential diagnosis for PCSM-LPD is very broad, including but not limited to mycosis fungoides, follicular lymphoma, angioimmunoblastic lymphoma, peripheral T-cell lymphoma, cutaneous lymphoid hyperplasia, basal cell carcinoma, unspecified lymphoma. Mycosis fungoides, a pathology that has been shown in studies to be the most common cutaneous lymphoma, is a disease in which lymphocytes become malignant and have cutaneous manifestations. In this patient, a lack of plaques and patches coupled with no pruritis symptoms help us rule out mycosis fungoides, which most studies suggest is the most common cutaneous lymphoma [6,8]. Another common cutaneous lymphoma that PCSM-LPD can be mistaken for is follicular lymphoma, a type of non-Hodgkin lymphoma where abnormal B lymphocytes proliferate. The absence of lymphadenopathy allowed us to rule out this pathology. 

There are multiple options for accepted treatment of PCSM-LPD; the most effective methods supported by literature are intralesional steroids, surgical removal, and radiotherapy. Prognosis after treatment with these different methods has been shown to be excellent, with resolution rates between 90-100% [1,9]. In this case report, we evidenced the complete resolution with punch biopsy alone. We suspect the clinical resolution may be due to the benign and nonaggressive nature of this disease, which highlights how minimal therapy can produce favorable outcomes.

Overall, we present this almost textbook-like presentation of PCSM-LPD, which is becoming increasingly studied in literature and a diagnosis that should be considered in the differential of similar presenting diseases; a diagnosis clinicians should heavily consider in light of diagnostic discrepancies within the current literature. Although the lower prevalence of this condition may cause providers to dismiss the possibility of its presence completely, this condition should be further studied to identify any other possible unique manifestations, which can provide clinicians with more information to rule out the pathology or to make the diagnosis. 

## Conclusions

In this case report, we detail the workup and diagnosis of primary cutaneous CD4+ small and medium pleomorphic T-cell lymphoproliferative disorder, a rare condition that may actually be more common than previously understood. Luckily, PCSM-LPD is benign and usually has an excellent prognosis, up to clinical resolution, with minimal treatment as described in this report. The high resolution rates and availability of many treatment options for this diagnosis make it clear that a good prognosis is achievable, so despite the low prevalence, PCSM-LPD should be considered in differentials of dermatologists and pathologists. 
